# 1-[2-(2,4-Dichloro­benz­yloxy)-2-phenyl­ethyl]-1*H*-1,2,4-triazole

**DOI:** 10.1107/S1600536808039196

**Published:** 2008-11-29

**Authors:** Özden Özel Güven, Hakan Tahtacı, M. Nawaz Tahir, Tuncer Hökelek

**Affiliations:** a Zonguldak Karaelmas University, Department of Chemistry, 67100 Zonguldak, Turkey; b Sargodha University, Department of Physics, Sargodha, Pakistan; c Hacettepe University, Department of Physics, 06800 Beytepe, Ankara, Turkey

## Abstract

In the mol­ecule of the title compound, C_17_H_15_Cl_2_N_3_O, the triazole ring is oriented at dihedral angles of 9.24 (6) and 82.49 (6)°, respectively, with respect to the phenyl and dichloro­benzene rings. The dihedral angle between the dichloro­benzene and phenyl rings is 88.57 (5)°. An intra­molecular C—H⋯O contact results in the formation of a planar five-membered ring.

## Related literature

For general backgroud, see: Paulvannan *et al.* (2001 ▶); Godefroi *et al.* (1969 ▶); Özel Güven *et al.* (2007*a* ▶,*b* ▶); Wahbi *et al.* (1995 ▶). For related structures, see: Peeters *et al.* (1979 ▶); Freer *et al.* (1986 ▶); Özel Güven *et al.* (2008*a* ▶,*b* ▶,*c* ▶,*d* ▶,*e* ▶,*f* ▶).
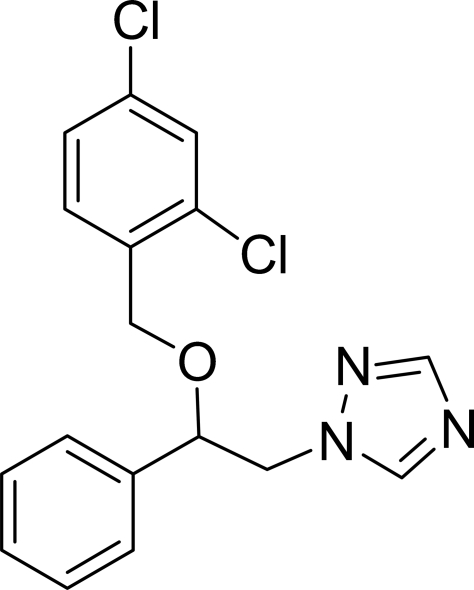


## Experimental

### 

#### Crystal data

C_17_H_15_Cl_2_N_3_O
*M* *_r_* = 348.22
Monoclinic, 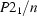

*a* = 10.5630 (3) Å
*b* = 13.7933 (5) Å
*c* = 11.4437 (4) Å
β = 101.840 (2)°
*V* = 1631.86 (10) Å^3^
*Z* = 4
Mo *K*α radiation
μ = 0.41 mm^−1^
*T* = 296 (2) K
0.35 × 0.25 × 0.15 mm

#### Data collection

Bruker Kappa APEXII CCD diffractometer
Absorption correction: multi-scan (*SADABS*; Bruker, 2005 ▶) *T*_min_ = 0.871, *T*_max_ = 0.942
17794 measured reflections
4055 independent reflections
3135 reflections with *I* > 2σ(*I*)
*R*_int_ = 0.026

#### Refinement

*R*[*F* ^2^ > 2σ(*F* ^2^)] = 0.037
*wR*(*F* ^2^) = 0.104
*S* = 1.04
4055 reflections
208 parameters
H-atom parameters constrained
Δρ_max_ = 0.32 e Å^−3^
Δρ_min_ = −0.24 e Å^−3^

### 

Data collection: *APEX2* (Bruker, 2007 ▶); cell refinement: *SAINT* (Bruker, 2007 ▶); data reduction: *SAINT*; program(s) used to solve structure: *SHELXS97* (Sheldrick, 2008 ▶); program(s) used to refine structure: *SHELXL97* (Sheldrick, 2008 ▶); molecular graphics: *ORTEP-3 for Windows* (Farrugia, 1997 ▶); software used to prepare material for publication: *WinGX* (Farrugia, 1999 ▶).

## Supplementary Material

Crystal structure: contains datablocks I, global. DOI: 10.1107/S1600536808039196/su2082sup1.cif

Structure factors: contains datablocks I. DOI: 10.1107/S1600536808039196/su2082Isup2.hkl

Additional supplementary materials:  crystallographic information; 3D view; checkCIF report

## Figures and Tables

**Table 1:** Hydrogen-bond geometry (Å, °)

*D*—H⋯*A*	*D*—H	H⋯*A*	*D*⋯*A*	*D*—H⋯*A*
C13—H13⋯O1	0.93	2.37	2.7191 (18)	102

Symmetry code: (i) 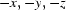.

**Fig. 1.**
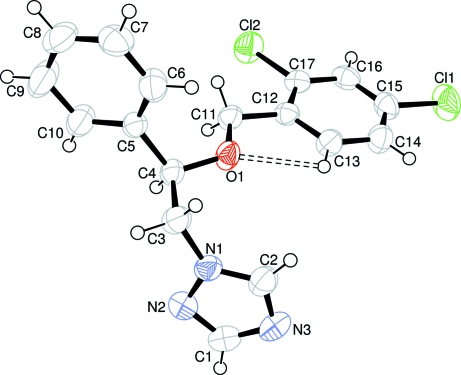
The molecular structure of the title compiund, with the atom-numbering scheme. Displacement ellipsoids are drawn at the 50% probability level. The intramolecular C-H···O contact is shown as a dashed line.

**Fig. 2.**
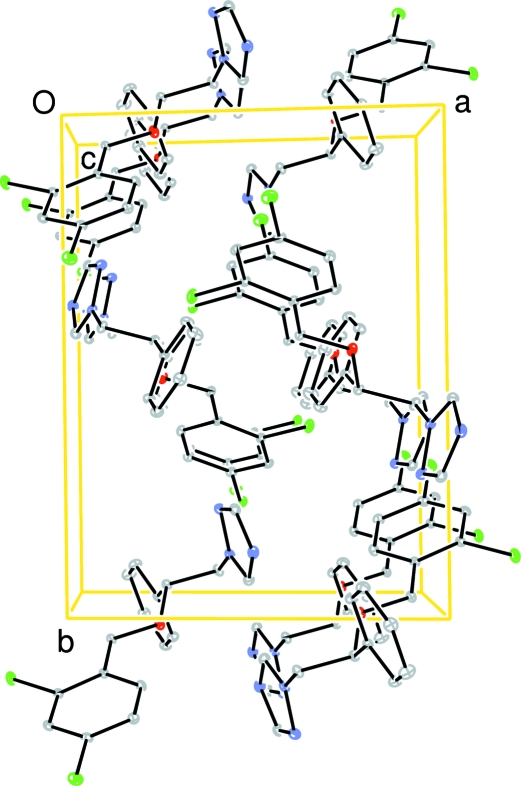
The crystal packing of the title compound, viewed along the c axis. Hydrogen atoms have been omitted for clarity.

## supplementary crystallographic information

### Comment

1,2,4-Triazoles are biologically interesting molecules and their chemistry is receiving considerable attention due to their antihypertensive, antifungal and antibacterial properties (Paulvannan *et al.*, 2001). Some ether structures containing the 1*H*-imidazole ring, like miconazole, econazole and sulconazole, have been synthesized and developed for clinical use as antifungal agents (Godefroi *et al.*, 1969). Also, antifungal activity of aromatic ethers possessing a 1*H*-1,2,4-triazole ring have been reported (Wahbi *et al.*, 1995). However, similar ether structures possessing a 1*H*-benzimidazole ring have been reported to show antibacterial activity more than antifungal activity (Özel Güven *et al.*, 2007*a*,b). The crystal structures of these ether derivatives, such as miconazole (Peeters *et al.*, 1979) and econazole (Freer *et al.*, 1986), have been reported. The crystal structures of 1*H*-benzimidazole ring containing ether derivatives (Özel Güven *et al.*, 2008*a*,b,c,d) and also, 1*H*-1,2,4-triazole ring containing ether derivative have been reported recently (Özel Güven *et al.*, 2008e). Here we report on the crystal structure of the 2,4-dichloro derivative of a 1*H*-1,2,4-triazole ring compound containing an ether structure.

In the molecule of the title compound (Fig. 1) the bond lengths and angles are generally within normal ranges. The planar triazole ring is oriented with respect to the phenyl and dichlorobenzene rings at dihedral angles of 9.24 (6)° and 82.49 (6)°, respectively. The dichlorobenzene ring is oriented with respect to the phenyl ring at a dihedral angle of 88.57 (5)°. The intramolecular C—H···O contact results in the formation of a planar five-membered ring (O1/H13/C11—C13), which is oriented with respect to dichlorobenzene ring at a dihedral angle of 0.65 (4)°, hence they are coplanar.

In the crystal structure of the title compound, the molecules stack along the *c* direction (Fig. 2). There is a weak intermolecular C—H···π contact between the methylene group and the dichlorobenzene ring [Table 1; where Cg3 is the centroid of the ring (C12-C17)].

### Experimental

The title compound was synthesized by the reaction of 1-phenyl-2-(1*H*-1,2,4 -triazol-1-yl)ethanol (Özel Güven *et al.*, 2008f) with NaH and the appropriate benzyl halide. To the solution of the alcohol (300 mg, 1.586 mmol) in DMF (4 ml) was added NaH (63 mg, 1.586 mmol) in small fractions. The appropriate benzyl halide (310 mg, 1.586 mmol) was added dropwise. The mixture was stirred at room temperature for 3 h, and excess hydride was decomposed with methyl alcohol (5 ml). After evaporation to dryness under reduced pressure, the crude residue was suspended with water and extracted with methylene chloride. The organic layer was dried over anhydrous sodium sulfate and then evaporated to dryness. The crude residue was purified by chromatography on a silica-gel column using chloroform as eluent. Crystals of the title compound, suitable for X-ray analysis, were obtained by recrystallization of the ether from 2-propanol (yield; 365 mg, 66%).

### Refinement

H atoms were positioned geometrically and constrained to ride on their parent atoms: C—H = 0.93 - 0.98 Å with *U*_iso_(H) = 1.2*U*_eq_(C).

### Crystal data

**Table:**

C_17_H_15_Cl_2_N_3_O	*F*_000_ = 720
*M**_r_* = 348.22	*D*_x_ = 1.417 Mg m^−^^3^
Monoclinic, *P*2_1_/*n*	Mo *K*α radiation λ = 0.71073 Å
Hall symbol: -P 2yn	Cell parameters from 1196 reflections
*a* = 10.5630 (3) Å	θ = 2.3–28.3º
*b* = 13.7933 (5) Å	µ = 0.41 mm^−^^1^
*c* = 11.4437 (4) Å	*T* = 296 (2) K
β = 101.840 (2)º	Rod-shaped, colorless
*V* = 1631.86 (10) Å^3^	0.35 × 0.25 × 0.15 mm
*Z* = 4	

### Data collection

**Table:**

Bruker Kappa APEXII CCD diffractometer	4055 independent reflections
Radiation source: fine-focus sealed tube	3135 reflections with *I* > 2σ(*I*)
Monochromator: graphite	*R*_int_ = 0.026
Detector resolution: 7.40 pixels mm^-1^	θ_max_ = 28.3º
*T* = 296(2) K	θ_min_ = 2.3º
ω scans	*h* = −14→14
Absorption correction: multi-scan(SADABS; Bruker, 2005)	*k* = −18→18
*T*_min_ = 0.871, *T*_max_ = 0.942	*l* = −15→14
17794 measured reflections	

### Refinement

**Table:**

Refinement on *F*^2^	Secondary atom site location: difference Fourier map
Least-squares matrix: full	Hydrogen site location: inferred from neighbouring sites
*R*[*F*^2^ > 2σ(*F*^2^)] = 0.037	H-atom parameters constrained
*wR*(*F*^2^) = 0.104	*w* = 1/[σ^2^(*F*_o_^2^) + (0.0424*P*)^2^ + 0.5043*P*] where *P* = (*F*_o_^2^ + 2*F*_c_^2^)/3
*S* = 1.04	(Δ/σ)_max_ < 0.001
4055 reflections	Δρ_max_ = 0.32 e Å^−^^3^
208 parameters	Δρ_min_ = −0.24 e Å^−^^3^
Primary atom site location: structure-invariant direct methods	Extinction correction: none

### Special details

**Table:**

Geometry. All e.s.d.'s (except the e.s.d. in the dihedral angle between two l.s. planes) are estimated using the full covariance matrix. The cell e.s.d.'s are taken into account individually in the estimation of e.s.d.'s in distances, angles and torsion angles; correlations between e.s.d.'s in cell parameters are only used when they are defined by crystal symmetry. An approximate (isotropic) treatment of cell e.s.d.'s is used for estimating e.s.d.'s involving l.s. planes.
Refinement. Refinement of *F*^2^ against ALL reflections. The weighted *R*-factor *wR* and goodness of fit *S* are based on *F*^2^, conventional *R*-factors *R* are based on *F*, with *F* set to zero for negative *F*^2^. The threshold expression of *F*^2^ > σ(*F*^2^) is used only for calculating *R*-factors(gt) *etc*. and is not relevant to the choice of reflections for refinement. *R*-factors based on *F*^2^ are statistically about twice as large as those based on *F*, and *R*- factors based on ALL data will be even larger.

### Fractional atomic coordinates and isotropic or equivalent isotropic displacement parameters (Å^2^)

**Table:**

	*x*	*y*	*z*	*U*_iso_*/*U*_eq_	
Cl1	0.52852 (6)	0.29276 (4)	−0.26180 (4)	0.07651 (18)	
Cl2	0.34215 (4)	0.12684 (4)	0.09114 (5)	0.06649 (16)	
O1	0.74240 (10)	0.02400 (8)	0.22002 (9)	0.0471 (3)	
N1	0.93446 (12)	−0.11684 (10)	0.21096 (11)	0.0455 (3)	
N2	0.90960 (15)	−0.21005 (11)	0.17678 (14)	0.0566 (4)	
N3	0.99426 (14)	−0.12858 (12)	0.04041 (13)	0.0576 (4)	
C1	0.94814 (17)	−0.21219 (14)	0.07456 (16)	0.0558 (4)	
H1	0.9437	−0.2683	0.0288	0.067*	
C2	0.98325 (16)	−0.07037 (14)	0.12863 (15)	0.0541 (4)	
H2	1.0065	−0.0052	0.1329	0.065*	
C3	0.90367 (16)	−0.08020 (14)	0.32066 (13)	0.0508 (4)	
H3A	0.9575	−0.0242	0.3472	0.061*	
H3B	0.9237	−0.1296	0.3820	0.061*	
C4	0.76226 (14)	−0.05147 (12)	0.30597 (12)	0.0420 (3)	
H4	0.7075	−0.1069	0.2751	0.050*	
C5	0.73484 (14)	−0.02155 (11)	0.42542 (12)	0.0399 (3)	
C6	0.77537 (18)	0.06624 (13)	0.47606 (15)	0.0557 (4)	
H6	0.8178	0.1096	0.4351	0.067*	
C7	0.7537 (2)	0.09083 (14)	0.58750 (16)	0.0615 (5)	
H7	0.7809	0.1507	0.6207	0.074*	
C8	0.6926 (2)	0.02748 (16)	0.64868 (17)	0.0668 (5)	
H8	0.6784	0.0440	0.7237	0.080*	
C9	0.6523 (2)	−0.06017 (17)	0.59975 (18)	0.0803 (7)	
H9	0.6113	−0.1037	0.6418	0.096*	
C10	0.6723 (2)	−0.08442 (14)	0.48764 (16)	0.0612 (5)	
H10	0.6431	−0.1438	0.4541	0.073*	
C11	0.61158 (14)	0.05336 (12)	0.18708 (13)	0.0423 (3)	
H11A	0.5879	0.0904	0.2514	0.051*	
H11B	0.5560	−0.0032	0.1725	0.051*	
C12	0.59372 (13)	0.11430 (10)	0.07624 (12)	0.0384 (3)	
C13	0.69449 (15)	0.13512 (12)	0.01952 (13)	0.0462 (4)	
H13	0.7767	0.1113	0.0512	0.055*	
C14	0.67532 (17)	0.19065 (13)	−0.08330 (14)	0.0516 (4)	
H14	0.7441	0.2042	−0.1201	0.062*	
C15	0.55413 (17)	0.22549 (12)	−0.13037 (13)	0.0493 (4)	
C16	0.45097 (16)	0.20677 (11)	−0.07723 (14)	0.0472 (4)	
H16	0.3689	0.2305	−0.1097	0.057*	
C17	0.47304 (14)	0.15158 (11)	0.02584 (13)	0.0414 (3)	

### Atomic displacement parameters (Å^2^)

**Table:**

	*U*^11^	*U*^22^	*U*^33^	*U*^12^	*U*^13^	*U*^23^
Cl1	0.0977 (4)	0.0735 (3)	0.0540 (3)	0.0099 (3)	0.0054 (2)	0.0273 (2)
Cl2	0.0427 (2)	0.0853 (4)	0.0740 (3)	0.0131 (2)	0.0178 (2)	0.0169 (2)
O1	0.0397 (5)	0.0633 (7)	0.0391 (5)	0.0109 (5)	0.0102 (4)	0.0171 (5)
N1	0.0429 (7)	0.0529 (8)	0.0419 (7)	0.0123 (6)	0.0114 (5)	0.0047 (6)
N2	0.0617 (9)	0.0497 (8)	0.0615 (9)	0.0087 (7)	0.0194 (7)	0.0056 (7)
N3	0.0511 (8)	0.0718 (10)	0.0559 (8)	0.0001 (7)	0.0246 (7)	−0.0063 (7)
C1	0.0504 (9)	0.0594 (11)	0.0600 (10)	0.0083 (8)	0.0170 (8)	−0.0083 (8)
C2	0.0532 (9)	0.0570 (10)	0.0563 (10)	−0.0027 (8)	0.0214 (8)	−0.0003 (8)
C3	0.0498 (9)	0.0656 (11)	0.0367 (7)	0.0176 (8)	0.0081 (6)	0.0062 (7)
C4	0.0447 (8)	0.0478 (8)	0.0340 (7)	0.0067 (6)	0.0093 (6)	0.0057 (6)
C5	0.0413 (7)	0.0441 (8)	0.0342 (7)	0.0067 (6)	0.0074 (6)	0.0038 (6)
C6	0.0672 (11)	0.0508 (10)	0.0492 (9)	−0.0085 (8)	0.0125 (8)	0.0027 (7)
C7	0.0751 (12)	0.0524 (10)	0.0533 (10)	0.0016 (9)	0.0043 (9)	−0.0124 (8)
C8	0.0790 (13)	0.0802 (14)	0.0453 (9)	0.0048 (11)	0.0223 (9)	−0.0111 (9)
C9	0.1129 (18)	0.0828 (15)	0.0574 (11)	−0.0245 (13)	0.0460 (12)	−0.0075 (11)
C10	0.0840 (13)	0.0552 (10)	0.0509 (9)	−0.0154 (9)	0.0291 (9)	−0.0069 (8)
C11	0.0389 (7)	0.0511 (9)	0.0378 (7)	0.0090 (6)	0.0103 (6)	0.0060 (6)
C12	0.0407 (7)	0.0402 (8)	0.0334 (6)	0.0047 (6)	0.0055 (5)	−0.0018 (6)
C13	0.0413 (8)	0.0567 (9)	0.0402 (8)	0.0082 (7)	0.0073 (6)	0.0060 (7)
C14	0.0535 (9)	0.0578 (10)	0.0448 (8)	0.0019 (7)	0.0128 (7)	0.0085 (7)
C15	0.0646 (10)	0.0430 (9)	0.0373 (7)	0.0048 (7)	0.0033 (7)	0.0056 (6)
C16	0.0493 (9)	0.0432 (8)	0.0442 (8)	0.0108 (7)	−0.0014 (7)	−0.0013 (6)
C17	0.0405 (7)	0.0412 (8)	0.0418 (7)	0.0050 (6)	0.0064 (6)	−0.0037 (6)

### Geometric parameters (Å, °)

**Table:**

Cl1—C15	1.7404 (16)	C6—H6	0.9300
Cl2—C17	1.7351 (16)	C7—H7	0.9300
O1—C4	1.4178 (17)	C8—C7	1.362 (3)
O1—C11	1.4148 (17)	C8—C9	1.363 (3)
N1—N2	1.354 (2)	C8—H8	0.9300
N1—C2	1.328 (2)	C9—C10	1.383 (2)
N1—C3	1.4508 (19)	C9—H9	0.9300
N3—C1	1.341 (2)	C10—H10	0.9300
N3—C2	1.314 (2)	C11—H11A	0.9700
C1—N2	1.315 (2)	C11—H11B	0.9700
C1—H1	0.9300	C12—C11	1.5010 (19)
C2—H2	0.9300	C12—C13	1.386 (2)
C3—H3A	0.9700	C12—C17	1.386 (2)
C3—H3B	0.9700	C13—C14	1.384 (2)
C4—C3	1.521 (2)	C13—H13	0.9300
C4—H4	0.9800	C14—H14	0.9300
C5—C4	1.5117 (19)	C15—C14	1.370 (2)
C5—C6	1.373 (2)	C15—C16	1.377 (2)
C5—C10	1.375 (2)	C16—H16	0.9300
C6—C7	1.383 (2)	C17—C16	1.383 (2)
			
C11—O1—C4	113.10 (11)	C7—C8—C9	119.91 (17)
N2—N1—C3	121.08 (14)	C7—C8—H8	120.0
C2—N1—N2	109.51 (14)	C9—C8—H8	120.0
C2—N1—C3	129.37 (15)	C8—C9—C10	120.08 (19)
C1—N2—N1	101.60 (14)	C8—C9—H9	120.0
C2—N3—C1	101.94 (14)	C10—C9—H9	120.0
N2—C1—N3	115.92 (16)	C5—C10—C9	120.59 (18)
N2—C1—H1	122.0	C5—C10—H10	119.7
N3—C1—H1	122.0	C9—C10—H10	119.7
N1—C2—H2	124.5	O1—C11—C12	109.39 (11)
N3—C2—N1	111.02 (16)	O1—C11—H11A	109.8
N3—C2—H2	124.5	O1—C11—H11B	109.8
N1—C3—C4	112.62 (13)	C12—C11—H11A	109.8
N1—C3—H3A	109.1	C12—C11—H11B	109.8
N1—C3—H3B	109.1	H11A—C11—H11B	108.2
C4—C3—H3A	109.1	C13—C12—C11	122.44 (13)
C4—C3—H3B	109.1	C17—C12—C11	120.33 (13)
H3A—C3—H3B	107.8	C17—C12—C13	117.22 (13)
O1—C4—C3	105.67 (12)	C12—C13—H13	119.3
O1—C4—C5	113.48 (12)	C14—C13—C12	121.37 (14)
O1—C4—H4	109.3	C14—C13—H13	119.3
C3—C4—H4	109.3	C13—C14—H14	120.3
C5—C4—C3	109.70 (12)	C15—C14—C13	119.37 (15)
C5—C4—H4	109.3	C15—C14—H14	120.3
C6—C5—C4	121.44 (14)	C14—C15—Cl1	119.59 (14)
C6—C5—C10	118.65 (15)	C14—C15—C16	121.40 (14)
C10—C5—C4	119.86 (14)	C15—C16—C17	118.01 (14)
C5—C6—C7	120.62 (17)	C15—C16—H16	121.0
C5—C6—H6	119.7	C16—C15—Cl1	119.00 (13)
C7—C6—H6	119.7	C17—C16—H16	121.0
C6—C7—H7	119.9	C12—C17—Cl2	119.60 (12)
C8—C7—C6	120.13 (17)	C16—C17—Cl2	117.76 (12)
C8—C7—H7	119.9	C16—C17—C12	122.62 (14)
			
C11—O1—C4—C5	−65.39 (16)	C6—C5—C10—C9	−0.9 (3)
C11—O1—C4—C3	174.37 (13)	C5—C6—C7—C8	0.5 (3)
C4—O1—C11—C12	−166.09 (12)	C9—C8—C7—C6	−0.2 (3)
C2—N1—N2—C1	0.78 (17)	C7—C8—C9—C10	−0.6 (4)
C3—N1—N2—C1	178.59 (14)	C8—C9—C10—C5	1.2 (4)
N2—N1—C2—N3	−0.86 (19)	C13—C12—C11—O1	0.3 (2)
C3—N1—C2—N3	−178.43 (14)	C17—C12—C11—O1	179.72 (13)
N2—N1—C3—C4	−81.93 (18)	C11—C12—C13—C14	179.31 (15)
C2—N1—C3—C4	95.4 (2)	C17—C12—C13—C14	−0.1 (2)
C1—N3—C2—N1	0.51 (19)	C11—C12—C17—Cl2	−0.4 (2)
C2—N3—C1—N2	0.0 (2)	C11—C12—C17—C16	−178.91 (14)
N3—C1—N2—N1	−0.5 (2)	C13—C12—C17—Cl2	179.09 (12)
O1—C4—C3—N1	−61.74 (18)	C13—C12—C17—C16	0.5 (2)
C5—C4—C3—N1	175.58 (14)	C12—C13—C14—C15	−0.3 (3)
C6—C5—C4—O1	−42.85 (19)	Cl1—C15—C14—C13	−178.35 (13)
C6—C5—C4—C3	75.08 (19)	C16—C15—C14—C13	0.3 (3)
C10—C5—C4—O1	139.60 (16)	Cl1—C15—C16—C17	178.75 (12)
C10—C5—C4—C3	−102.47 (18)	C14—C15—C16—C17	0.1 (2)
C4—C5—C6—C7	−177.53 (16)	Cl2—C17—C16—C15	−179.10 (12)
C10—C5—C6—C7	0.0 (3)	C12—C17—C16—C15	−0.5 (2)
C4—C5—C10—C9	176.68 (18)		

### Hydrogen-bond geometry (Å, °)

**Table:**

*D*—H···*A*	*D*—H	H···*A*	*D*···*A*	*D*—H···*A*
C13—H13···O1	0.93	2.37	2.7191 (18)	102
